# A pooled analysis of the prognostic value of PD-L1 in melanoma: evidence from 1062 patients

**DOI:** 10.1186/s12935-020-01187-x

**Published:** 2020-03-30

**Authors:** Jing Yang, Meilian Dong, Yifang Shui, Yue Zhang, Zhigang Zhang, Yin Mi, Xiaoxiao Zuo, Li Jiang, Ke Liu, Zheyan Liu, Xiaobin Gu, Yonggang Shi

**Affiliations:** 1 Department of Radiation Oncology, The First Affiliated Hospital of Zhengzhou University, Zhengzhou, 450000 Henan People’s Republic of China; 2 Department of Breast Surgery, The First Affiliated Hospital of Zhengzhou University, Zhengzhou, 450000 Henan People’s Republic of China

**Keywords:** PD-L1, Meta-analysis, Prognosis, Melanoma, Risk factor

## Abstract

**Background:**

Programmed death-ligand 1 (PD-L1) was the first identified ligand of programmed death-1 (PD-1). PD-1/PD-L1 interactions inhibit T cell-mediated immune responses, limit cytokine production, and promote tumor immune escape. Recently, many studies have investigated the prognostic value of PD-L1 expression in patients with melanoma. However, the results of these analyses remain a subject of debate. We have therefore carried out a meta-analysis to identify the prognostic role of PD-L1 in melanoma.

**Methods:**

A thorough medical literature search was performed in the databases PubMed, Web of Science, and Embase until October 2019. The pooled hazard ratios (HRs) and 95% confidence intervals (95% CIs) were calculated to evaluate the correlation between PD-L1 overexpression and prognosis. Publication bias was evaluated using Begg’s test and Egger’s test.

**Results:**

Thirteen articles with 1062 enrolled patients were included in this meta-analysis. High PD-L1 expression did not correlate with overall survival (OS) (HR = 0.93, 95% CI 0.57–1.52, P = 0.781) or progression-free survival (PFS) (HR = 0.82, 95% CI 0.43–1.54, P = 0.535). However, PD-L1 overexpression correlated with the absence of lymph node (LN) metastasis (OR = 0.46, 95% CI 0.22–0.95, P = 0.036). Further, there was no significant relationship between PD-L1 expression and sex (OR = 1.29, 95% CI 0.90–1.84, P = 0.159), age (OR = 0.90, 95% CI 0.51–1.57, P = 0.708), or Eastern Cooperative Oncology Group Performance Status (OR = 0.55, 95% CI 0.06–4.83, P = 0.592).

**Conclusions:**

This meta-analysis suggested that PD-L1 expression did not predict an inferior prognosis in patients with melanoma. However, high PD-L1 expression was associated with absence of LN metastasis in such patients.

## Background

Melanoma is the most fatal form of skin cancer, and the incidence rates continue to increase dramatically [1]. Worldwide, approximately 232,100 new cases of cutaneous melanoma are diagnosed each year, and 55,500 patients die annually [2]. Ultraviolet exposure, skin type, indoor tanning, and a personal history of prior melanoma are risk factors of melanoma [3–5]. The most important prognostic factor of melanoma is the *BRAF* mutational status [6]. The other prognostic factors are American Joint Committee on Cancer (AJCC) melanoma TNM (tumor, node, metastasis) staging [7], Clark level, and Breslow thickness [8], and they are useful for the clinical management of patients with melanoma. In the United States, patients present melanoma at different stages, with 84% of them presenting localized disease, 9% presenting regional disease, and 4% exhibiting distant metastasis [9]. The prognosis for patients with localized disease is promising, with a 5-year survival rate of over 90% [10]. Whereas the prognosis for patients with unresectable stage III–IV tumors is poor, as the 10‐year overall survival (OS) is only 10% to 15% for those patients [1]. In recent years, significant progress has been achieved in the development of targeted therapies and immunotherapy [11, 12]; however, novel prognostic markers are still needed for tailoring personal treatment strategies.

In recent years, immune inhibitory signaling pathways have been recognized to play a pivotal role in the maintenance of an immunosuppressive microenvironment that favors cancer development [13]. One important co-inhibitory pathway is the programmed death-ligand 1 (PD-L1) and programmed death-1 (PD-1) axis [14]. PD-1 is expressed in a wide range of immune cells, and its expression is induced on effector T‐cells in response to inflammatory signals [15]. PD-L1 (also known as B7-H1 or CD274) was the first identified ligand of PD-1 [15, 16]. PD-L1 is also widely expressed in various cell types including lymphocytes, vascular endothelium, mesenchymal stem cells, neuronal cells, and tumor cells [15]. PD-1/PD-L1 interactions inhibit T-cell-mediated immune responses, limit cytokine production, and promote tumor immune escape [17]. Recent studies have also demonstrated that tumor-derived extracellular vesicles (EVs) act as messengers of intercellular communication [18]. Exosomal microRNAs (miRNAs), which are transferred by EVs, are promising and reliable tools for cancer diagnosis and clinical application [18]. PD-L1 overexpression has been examined as a prognostic factor in diverse cancers including lung cancer [19], gastric cancer [20], ovarian cancer [21], breast cancer [22], prostate cancer [23], bladder cancer [24], cervical cancer [25], cholangiocarcinoma [26], colorectal cancer [27], nasopharyngeal carcinoma [28], diffuse large B-cell lymphoma [29], pancreatic cancer [30], soft-tissue sarcoma [31], renal cell carcinoma [32], and head and neck squamous cell carcinoma [33]. In addition, in patients with melanoma, exosomal PD-L1 is an indicator of immune activation early after the initiation of treatment with immune checkpoint inhibitors (ICIs) and is associated with clinical response to ICIs [34].

Previous studies have also assessed the prognostic value of PD-L1 expression in patients with melanoma [35–47]; however, the results remain controversial. We have therefore performed a meta-analysis to assess whether PD-L1 expression was associated with prognosis and clinicopathological factors in patients with melanoma.

## Materials and methods

### Search strategy

We carried out the meta-analysis in accordance with the preferred reporting items for systematic reviews and meta-analyses (PRISMA) guidelines [48]. We comprehensively searched the databases PubMed, Web of Science, and Embase using the following keywords: (PD-L1 OR B7-H1 OR programmed cell death 1 ligand 1 OR CD274) AND (melanoma OR malignant melanoma) AND (survival OR prognostic OR prognosis OR outcome). We searched articles until October 2019. The reference lists were also carefully checked to identify additional eligible studies. All analyses were performed using the data of previously published studies. Therefore, no ethical approval or patient consent was required for this study.

### Selection criteria

Studies were included if they met the following criteria: (1) inclusion of patients diagnosed with histologically confirmed melanoma; (2) detection of PD-L1 expression in the melanoma tissue using immunohistochemistry (IHC) studies; (3) identification of a definite cut-off value to determine PD-L1 overexpression; (4) reporting a correlation between PD-L1 and survival including OS and/or progression-free survival (PFS), or providing sufficient information to compute the hazard ratio (HR) and 95% confidence interval (95% CI); (5) published in English language. The exclusion criteria were as follows: (1) case reports, reviews, letters, and correspondences; (2) studies without available or usable information; (3) studies lacking survival data; (4) animal studies; (5) non-English articles; (6) duplicate studies.

### Data extraction and quality assessment

Two researchers (JY and MD) independently extracted basic information from the included studies, and any disagreements were resolved by discussion with a third researcher (XG). The following data were extracted from eligible studies: the first author’s name, publication year, ethnicity of patients, sample size, age, tumor stage, study period, sampling specimen, detection method, treatment, cut-off values, methods of survival analysis, follow-up time, and clinicopathologic parameters. When both univariate and multivariate analyses of OS and/or PFS were conducted in the included studies, we extracted the data of multivariate analysis. The results of univariate analysis were adopted when only the univariate analysis was performed. The quality of each eligible study was evaluated using the Newcastle–Ottawa Scale (NOS) [49]. The NOS scale consists of three items describing the study quality: selection (0–4 points), comparability (0–2 points), and outcome assessment (0–3 points). The maximum NOS score is 9 points, and studies with a score of 6 points or higher are considered high-quality studies.

### Statistical analysis

The pooled HRs and 95% CIs were calculated to evaluate the correlation between PD-L1 overexpression and prognosis (OS and PFS). The association between PD-L1 expression and clinicopathological parameters were assessed by combining the odds ratios (ORs) and their 95% CIs. Cochrane’s Q test and *I*^2^ metric were used to evaluate the statistical heterogeneity of the pooled data. A P value of less than 0.1 or an *I*^2^ value of more than 50% indicated significant heterogeneity, and a random effects model was employed for calculation. Otherwise, a fixed effects model was applied. Subgroup analyses were performed to detect sources of heterogeneity. Sensitivity analysis was carried out by omitting each individual study to examine the robustness of the results. The potential publication bias was assessed with Begg’s test and Egger’s test. All statistical analyses were performed using Stata version 12.0 (STATA Corp., College Station, TX). P < 0.05 was considered to indicate statistical significance.

## Results

### Literature selection

**Fig. 1:**
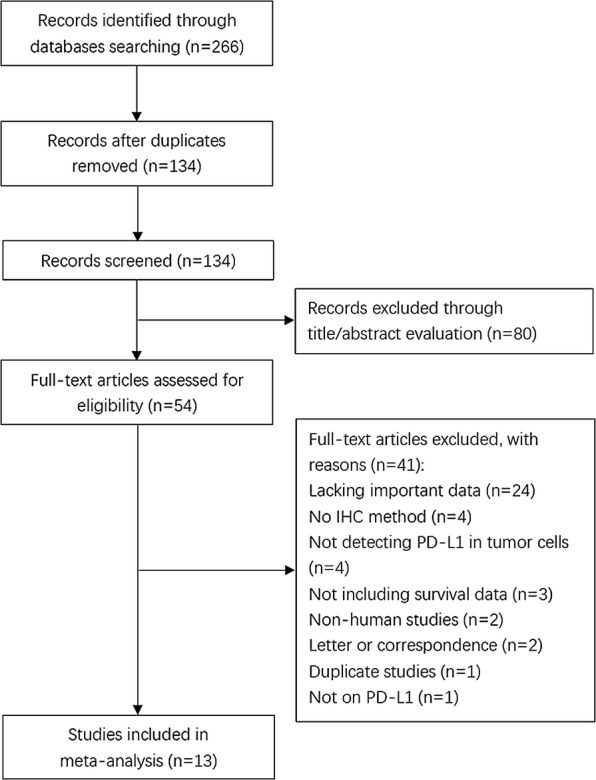
Flowchart of study screening and selection process

A total of 266 studies were identified by the primary search strategy. After removing duplicates, 134 studies were evaluated by title and abstract screening; 80 studies were then discarded. Thus, 54 articles remained for further full-text estimation. After careful reading of the full text, 41 studies were removed for the following reasons: 24 studies lacked necessary data, 4 studies did not apply the IHC method, 4 studies did not detect PD-L1 expression in tumor cells, 3 studies lacked survival data, 2 studies were non-human studies, 2 studies were letters or correspondences, 1 study was duplicated, and 1 study did not focus on PD-L1. Ultimately, 13 articles [35–47] were included in this meta-analysis. A flowchart of the literature selection procedure is shown in Fig. 1.

### Characteristics of studies

Detailed information of the included studies is given in Table 1. The included studies were published during 2011–2019 and from 7 countries. Three studies were conducted in the United States [37, 41, 42], 3 in China [43, 44, 47], 2 in Italy [38, 40], 2 in Germany [45, 46], 1 in Korea [35], 1 in The Netherlands [36], and 1 in Australia [39]. The total sample size was 1062 patients, ranging from 23 to 147 patients per paper, with a mean value of 81.7. All studies used IHC to detect PD-L1 expression in the tumor tissue. The cutoff values for PD-L1 expression differed by > 5%, > 1%, H-score > 5, and H-score > 1 in the included studies. Two studies had a prospective design [41, 45] and 11 studies were retrospective studies [35–40, 42–44, 46, 47]. Ten studies [36–38, 40–42, 44–47] and 8 studies [35, 37, 39, 40, 43–46] provided data on OS and PFS, respectively. Eight studies [35–38, 40–42, 45] enrolled patients with metastatic disease and 5 studies [39, 43, 44, 46, 47] recruited patients with the disease at mixed stages. These studies generally had high quality, with NOS scores ranging from 6 to 9.

**Table 1:** Basic characteristics of the studies included for meta-analysis

Study	Year	Country	Sample size	Ethnicity	Age	Stage	Treatment	Study duration	Sampling	Detection method	Survival outcomes	Types of analysis	Cut-off value	Study design	Follow-up (month)	NOS score
Cho	2016	Korea	37	Asian	58 (21–81)	Metastatic	ICIs	Jan-Dec, 2015	Tissue	IHC	PFS	Univariate	> 5%	Retrospective	6.4 (1.4–11.2)	8
Gadiot	2011	The Netherlands	63	Caucasian	53 (18–91)	Metastatic	Mixed	2000–2004	Tissue	IHC	OS	Univariate	> 1%	Retrospective	51.2 (6.8–134.8)	9
Johnson	2018	USA	142	Caucasian	63.4	Metastatic	ICIs	2010–2016	Tissue	IHC	OS, PFS	Univariate	> 5%	Retrospective	NA	8
Madonna	2018	Italy	114	Caucasian	61 (25–90)	Metastatic	ICIs	2010–2013	Tissue	IHC	OS	Univariate	> 5%	Retrospective	NA	7
Madore	2015	Australia	58	Caucasian	61	Mixed	Mixed	NA	Tissue	IHC	PFS	Univariate	> 1%	Retrospective	49.2 (4.1–229.2)	9
Massi	2015	Italy	80	Caucasian	56 (21–82)	Metastatic	BRAFi	2011–2014	Tissue	IHC	OS, PFS	Univariate	> 5%	Retrospective	9	7
Morrison	2018	USA	137	Caucasian	61	Metastatic	ICIs	1990–2016	Tissue	IHC	OS	Univariate	> 1%	Prospective	16.2	8
Obeid	2016	USA	147	Caucasian	58.9	Metastatic	Mixed	1982–2007	Tissue	IHC	OS	Univariate	> 5%	Retrospective	1–358	8
Ren	2018	China	78	Asian	61.5 (31–85)	Mixed	Surgery	2005–2012	Tissue	IHC	PFS	Multivariate	H-score > 5	Retrospective	73.5 (60–151)	8
Ren	2019	China	89	Asian	63 (40–90)	Mixed	Surgery	2010–2017	Tissue	IHC	OS, PFS	Univariate	> 5%	Retrospective	NA	7
Schaper-Gerhardt	2018	Germany	58	Caucasian	61 (27–88)	Metastatic	BRAFi	NA	Tissue	IHC	OS, PFS	Univariate	> 1%	Prospective	10.3 (0.6–36.3)	8
Thierauf	2015	Germany	23	Caucasian	66	Mixed	Surgery	NA	Tissue	IHC	OS, PFS	Univariate	> Score 1	Retrospective	NA	6
Wang	2019	China	36	Asian	48 (27–77)	Mixed	Mixed	2004–2018	Tissue	IHC	OS	Univariate	> 1%	Retrospective	NA	7

*PD-L1* programmed death ligand 1, *IHC* immunohistochemistry, *OS* overall survival, *PFS* progression-free survival, *NA* not available, *ICIs* immune checkpoint inhibitors, *BRAFi* BRAF inhibitor, *NOS* Newcastle–Ottawa Quality Assessment Scale

### Correlation between PD-L1 expression and OS

**Fig. 2:**
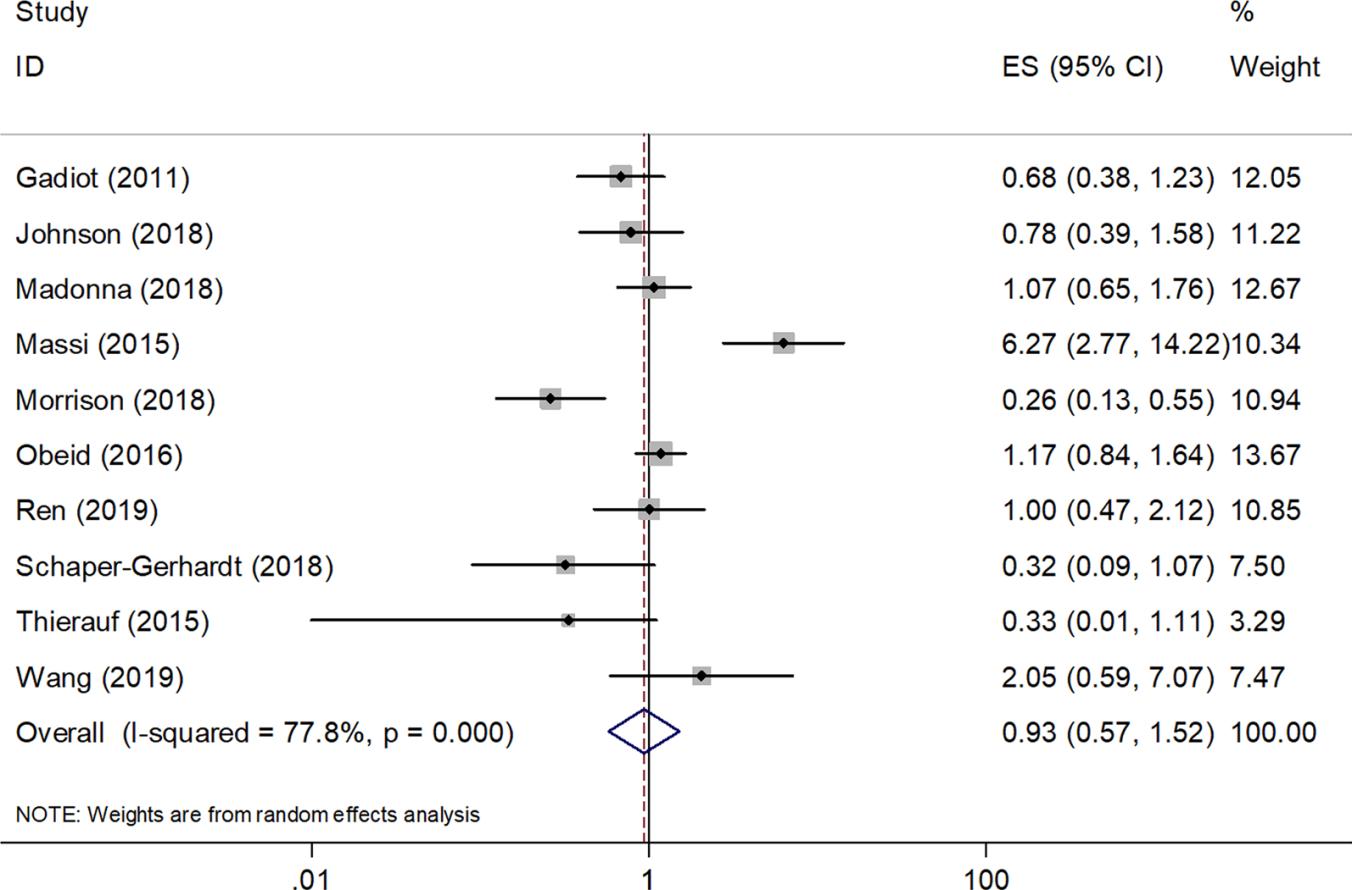
Forest plots of the association between PD-L1 expression and overall survival (OS) in melanoma

A total of 10 studies enrolling 889 patients [36–38, 40–42, 44–47] reported data on PD-L1 for the prognosis of OS. Because of significant heterogeneity among studies (*I*^2^ = 77.8%, P < 0.001), a random effects model was used. As shown in Fig. 2 and Table 2, the pooled results indicated a nonsignificant relationship between PD-L1 expression and OS (HR = 0.93, 95% CI 0.57–1.52, P = 0.781). We then performed subgroup analysis for further investigation. As shown in Table 2, PD-L1 overexpression was shown to have no significant prognostic role in OS in the subgroups stratified by ethnicity, stage, sample size, cut-off value, and treatment, with P > 0.05 in all subgroups.

**Table 2:** Subgroup analysis of association of PD-L1 expression and OS and PFS in melanoma

Factors	No. of studies	No. of patients	Effects model	HR (95% CI)	p	Heterogeneity	
						*I*^2^ (%)	P
Overall survival							
Total	10	889	Random	0.93 (0.57–1.52)	0.781	77.8	< 0.001
Ethnicity							
Asian	2	125	Fixed	1.21 (0.64–2.31)	0.553	0	0.335
Caucasian	8	764	Random	0.85 (0.48–1.52)	0.59	82.2	< 0.001
Stage							
Metastatic	7	741	Random	0.89 (0.49–1.61)	0.704	84.4	< 0.001
Mixed	3	148	Fixed	1.11 (0.60–2.06)	0.740	0	0.365
Sample size							
< 80	5	269	Fixed	0.77 (0.52–1.16)	0.211	27.1	0.241
≥ 80	5	620	Random	1.08 (0.52–2.27)	0.830	87.9	< 0.001
Cut–off value							
> 5%	5	572	Random	1.37 (0.80–2.36)	0.080	77.2	0.002
> 1% and others	5	317	Random	0.53 (0.26–1.08)	0.252	58.3	0.048
Treatment							
ICIs	3	393	Random	0.62 (0.27–1.40)	0.252	79.5	0.008
BRAFi	2	138	Random	1.47 (0.08–27.21)	0.797	93.6	< 0.001
Surgery	2	112	Fixed	0.91 (0.44–1.85)	0.666	0	0.380
Mixed	3	246	Fixed	1.06 (0.80–1.41)	0.789	44.7	0.164
Progression-free survival							
Total	8	565	Random	0.82 (0.43–1.54)	0.535	75.4	< 0.001
Ethnicity							
Asian	3	204	Fixed	0.93 (0.57–1.50)	0.756	44.9	0.163
Caucasian	5	361	Random	0.79 (0.32–2.08)	0.629	83.9	< 0.001
Stage							
Metastatic	4	317	Random	0.88 (0.26–3.03)	0.838	85.4	< 0.001
Mixed	4	248	Random	0.75 (0.40–1.42)	0.380	52.2	0.099
Sample size							
< 80	5	254	Random	0.60 (0.30–1.21)	0.153	50.6	0.088
≥ 80	3	311	Random	1.30 (0.42–4.01)	0.654	88.2	< 0.001
Cut-off value							
> 5%	4	348	Random	1.00 (0.34–2.90)	1	84.6	< 0.001
> 1% and others	4	217	Random	0.66 (0.32–1.39)	0.274	56	0.078
Treatment							
ICIs	2	179	Fixed	0.58 (0.30–1.13)	0.110	0	0.321
BRAFi	2	138	Random	1.56 (0.20–11.84)	0.668	91.5	0.001
Surgery	3	190	Random	0.74 (0.30–1.84)	0.515	67.2	0.047
Mixed	1	58	–	0.69 (0.30–1.59)	0.383	–	–

*ICIs* immune checkpoint inhibitors, *BRAFi* BRAF inhibitor

### Association between PD-L1 expression and PFS

**Fig. 3:**
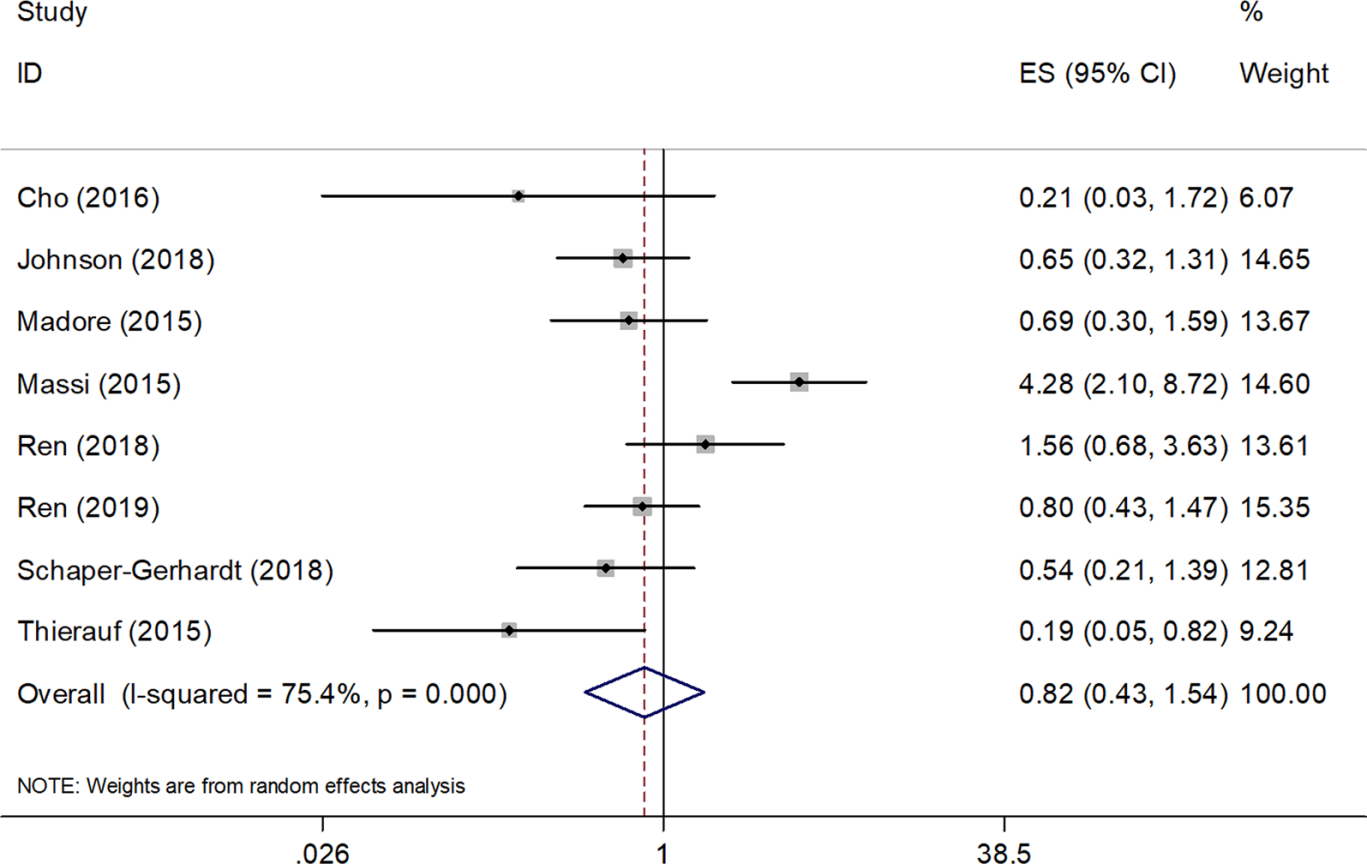
Forest plots of the association between PD-L1 expression and progression-free survival (PFS) in melanoma

Eight studies enrolling a total of 565 patients [35, 37, 39, 40, 43–46] were included in the PFS analysis. Pooled results revealed that elevated PD-L1 expression had no significant effect on PFS in melanoma (HR = 0.82, 95% CI 0.43–1.54, P = 0.535, Fig. 3, Table 2), and a random effects model was used for the significant heterogeneity (*I*^2^ = 75.4%, P < 0.001). The subgroup analysis indicated that PD-L1 did not predict PFS in different subgroups (Table 2).

### Relationship between PD-L1 and clinicopathological factors

**Fig. 4:**
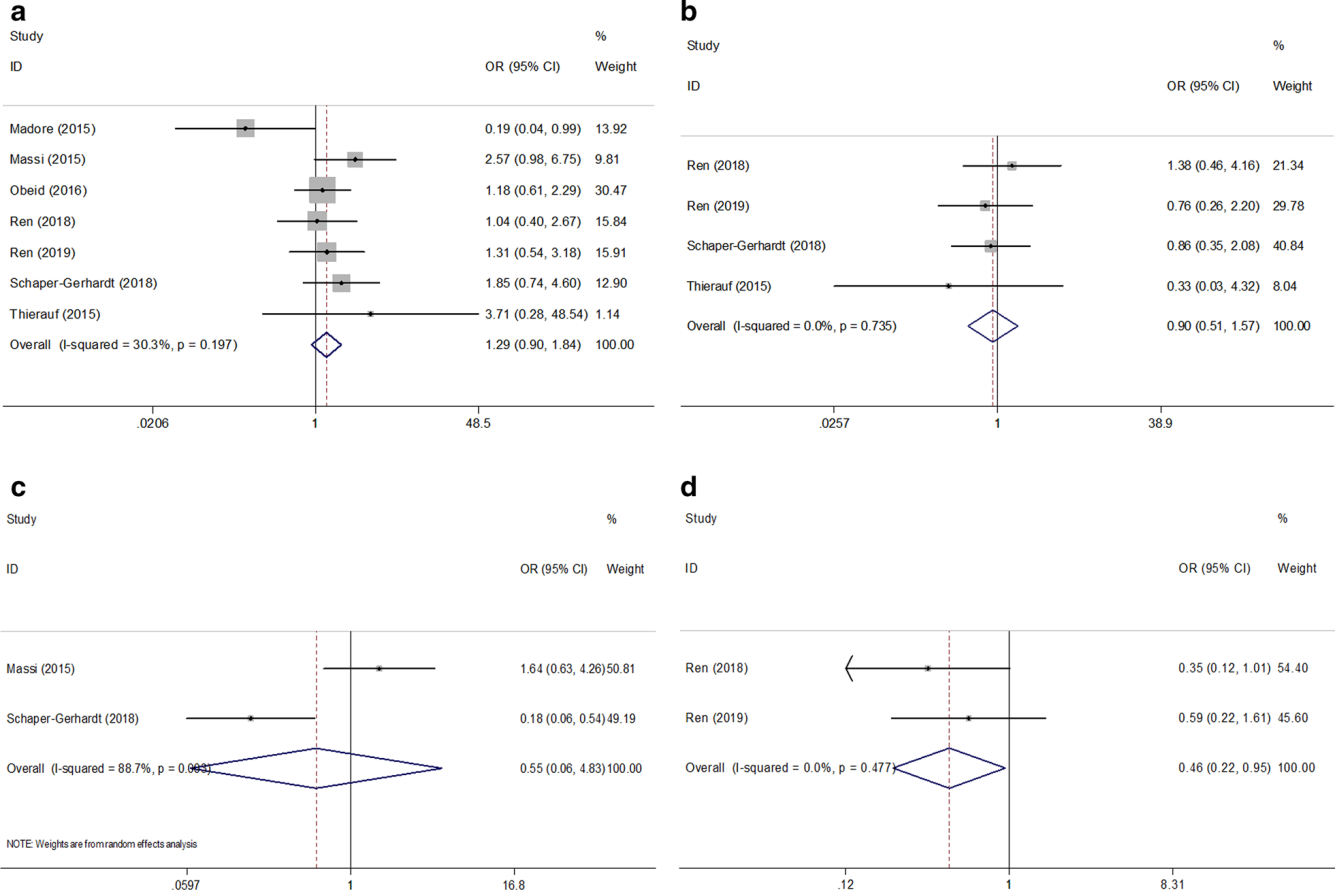
Forest plots of the association of high PD-L1 expression with clinicopathological factors: **a** sex; **b** age; **c** ECOG PS; and **d** LN metastasis

Using the available data of the included studies, the association between PD-L1 expression and 4 clinicopathological features was analyzed, namely, sex (male vs. female), age (≥ 60 vs. < 60, years), Eastern Cooperative Oncology Group Performance Status (ECOG PS) (≥ 1 vs. 0), and lymph node (LN) metastasis (yes vs. no). The data from 2 studies that enrolled 167 patients showed correlation of PD-L1 overexpression with the absence of LN metastasis (OR = 0.46, 95% CI 0.22–0.95, P = 0.036, Table 3, Fig. 4). However, there was no significant relationship between PD-L1 expression and sex (n = 7, OR = 1.29, 95% CI 0.90–1.84, P = 0.159), age (n = 4, OR = 0.90, 95% CI 0.51–1.57, P = 0.708), or ECOG PS (n = 2, OR = 0.55, 95% CI 0.06–4.83, P = 0.592, Table 3, Fig. 4).

**Table 3:** Meta-analysis of the association between PD-L1 expression and clinicopathological features of melanoma

Clinicopathological features	No. of studies	No. of patients	Effects model	OR (95% CI)	p	Heterogeneity	
						*I*^2^ (%)	Ph
Sex (male vs female)	7	533	Fixed	1.29 (0.90–1.84)	0.159	30.3	0.197
Age (≥ 60 vs < 60, years)	4	248	Fixed	0.90 (0.51–1.57)	0.708	0	0.735
ECOG PS (≥ 1 vs 0)	2	138	Random	0.55 (0.06–4.83)	0.592	88.7	0.003
LN metastasis (yes vs no)	2	167	Fixed	0.46 (0.22–0.95)	0.036	0	0.477

*ECOG PS* Eastern Cooperative Oncology Group performance status, *LN* lymph node

### Sensitivity analysis

**Fig. 5:**
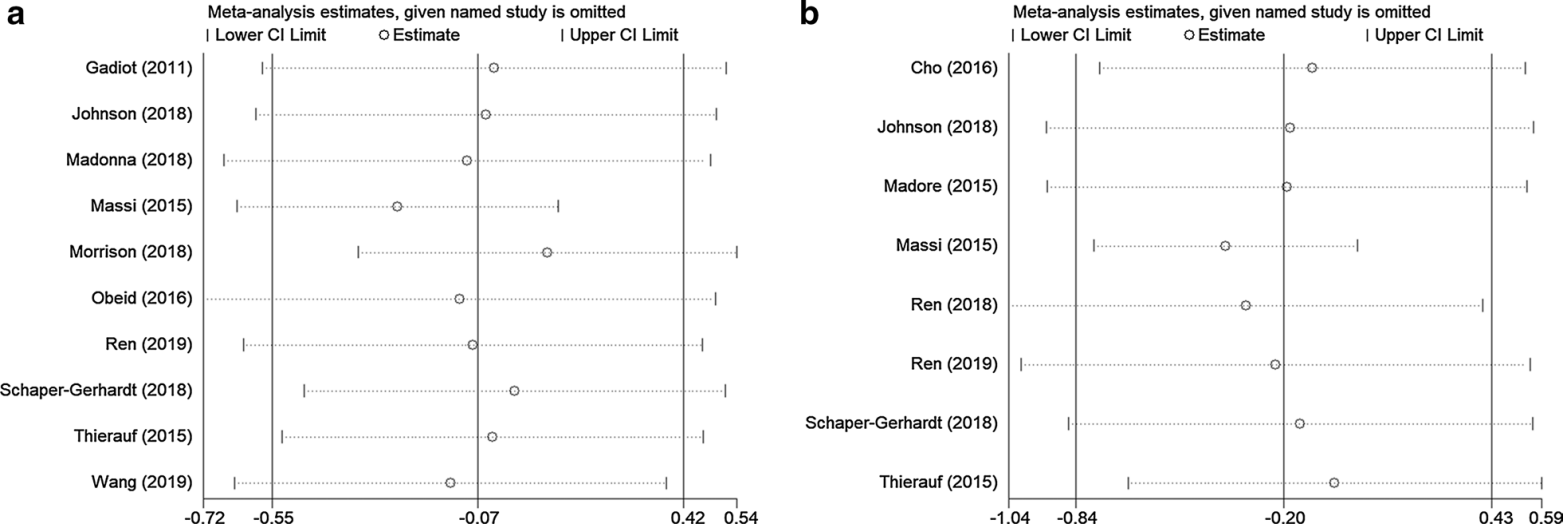
The sensitivity analysis of the meta‐analysis. **a** The sensitivity analysis for high PD-L1 expression with OS. **b** The sensitivity analysis for or high PD-L1 expression with PFS

Sensitivity analysis was conducted for OS and PFS (Fig. 5) by sequential omission of each study. As shown in Fig. 5, the overall results of OS and PFS were not substantially changed by deletion of any single study, indicating the credibility of the results.

### Publication bias

**Fig. 6:**
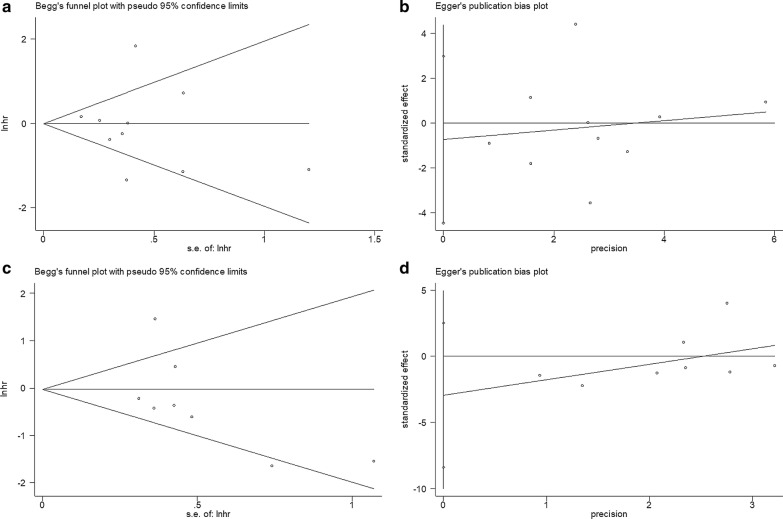
Publication bias tests. **a** Begg’s test for OS, p = 0.721; **b** Egger’s test for OS, p = 0.662; **c** Begg’s test for PFS, p = 0.108; and **d** Egger’s test for PFS, p = 0.235

Begg’s test and Egger’s test were adopted to determine whether potential publication bias existed in this meta-analysis. The funnel plots were symmetric (Fig. 6), and all of the P values of publication bias were more than 0.05 (Begg’s P = 0.721, Egger’s P = 0.662 for OS and Begg’s P = 0.108, Egger’s P = 0.235 for PFS, Fig. 6). These data suggested that there was no significant publication bias in this meta-analysis.

## Discussion

The association between PD-L1 expression and prognosis in melanoma has been explored extensively in previous studies; however, the results were inconsistent. The conflicting data from different studies promoted us to conduct the current meta-analysis by pooling data from 13 included studies, which were all strictly selected according to uniform inclusion and exclusion criteria. Our meta-analysis of studies that enrolled 1062 patients demonstrated that high PD-L1 expression was not associated with poor prognosis in patients with melanoma. In addition, PD-L1 expression remained a non-significant prognostic factor in various subgroups of OS and PFS. PD-L1 overexpression was found to be correlated with the absence of LN metastasis, although the predominant connection was based on the data of 2 studies. The results of this meta-analysis suggest that PD-L1 may be not be predictive of outcomes of melanoma management. To the best of our knowledge, the present study is the first meta-analysis investigating the prognostic significance of PD-L1 expression in melanoma.

Immune escape is essential for cancer development, progression, and resistance to therapy [13]. Lieping Chen et al. identified and cloned the human *B7*-*H1* gene in the year 1999 and found that this molecule could negatively regulate T cell function through the induction of IL-10 [16]. Accumulating evidence shows that PD-L1 plays a central role in the regulation of the immune responses in the tumor microenvironment [50]. PD-L1 binds to PD-1 and inhibits T cell proliferation and its cytokine secretion and leads to apoptosis, anergy, and exhaustion of T cells [51]. Therefore, blockade of the PD-1/PD-L1 interaction is an important therapeutic strategy for cancer. Tumor-intrinsic PD-L1 signals can enhance the ability of melanoma cells to proliferate and metastasize [52]. Melanoma has seen the broadest applications and superior responses to anti-PD-L1/PD-1 therapies [53]. Recent studies have demonstrated that anti-PD-L1 antibody induced durable tumor regression and prolonged stabilization of disease in patients with advanced cancer, including non-small cell lung cancer (NSCLC), melanoma, and colorectal cancer [54]. In addition, the combination of PD-1 and CTLA-4 blockade was more effective than either agent alone in metastatic melanoma [55]. Therefore, there is rationale to identify PD-L1 as a biomarker for assessing cancer therapeutic responses and survival outcomes in patients with melanoma. The findings of our meta-analysis indicate that PD-L1 may not be helpful in prognosis of melanoma, which may be validated in further large-scale prospective clinical trials.

Many previous studies have investigated the impact of PD-L1 on the prognosis of solid tumors through meta-analyses [56]. Iacovelli and colleagues conducted a meta-analysis of 6 studies and showed that increased PD-L1 expression was an independent prognostic factor in renal cell carcinoma [57]. Another meta-analysis also demonstrated that high PD-L1 expression was a poor prognostic biomarker in patients with non-Hodgkin lymphoma [58]. A meta-analysis of studies that enrolled 721 patients also confirmed the prognostic significance of PD-L1 expression in thyroid cancer [59]. However, some meta-analyses failed to identify a significant prognostic effect of PD-L1 in cancer. For example, Fan’s meta-analysis reported a non-significant relationship between PD-L1 expression and OS in NSCLC [60]. Moreover, a more recent study of 1060 patients indicated that PD-L1 overexpression did not correlate with the poor prognosis of patients with oral squamous cell carcinoma (OSCC) [61]. The results of the current meta-analysis in melanoma were in line with the findings of NSCLC and OSCC [60, 61].

Although this is the first meta-analysis of the association between PD-L1 and the prognosis of melanoma, some limitations need to be noted. First, the heterogeneity among studies cannot be ignored. Patient ethnicity, treatment, follow-up, and other factors could influence survival, which may have contributed to this heterogeneity. Second, the included studies used different monoclonal and polyclonal PD-L1 antibodies for IHC, and the cut-off values were not uniform. Third, all included studies were published in the English language, and absence of including studies published in non-English languages may lead to publication bias.

## Conclusions

In summary, this meta-analysis suggested that PD-L1 expression did not predict inferior prognosis in patients with melanoma. However, high PD-L1 expression was associated with absence of LN metastasis. Because of the limitations of our meta-analysis, further large-scale and prospective trials that use a uniform cut-off value of PD-L1 expression are needed to verify our results.

## Data Availability

The data that support the findings of this study are available from the corresponding author upon reasonable request.

## Abbreviations

PD-L1: Programmed death-ligand 1
PD-1: Programmed death-1
HR: Hazard ratio
CI: Confidence interval
OS: Overall survival
PFS: Progression-free survival
LN: Lymph node
AJCC: American Joint Committee on Cancer
TNM: Tumor, node, metastasis
IHC: Immunohistochemistry
EV: Extracellular vesicle
ICI: Immune checkpoint inhibitors
ECOG PS: Eastern Cooperative Oncology Group performance status
NSCLC: Non-small cell lung cancer
OSCC: Oral squamous cell carcinoma
PRISMA: Preferred reporting items for systematic reviews and meta-analyses
NOS: Newcastle–Ottawa Scale
OR: Odds ratio
